# Supplementary material for: Leucine Supplementation Improves Diastolic Function in HFpEF by HDAC4 Inhibition
Source: Cells. 2023 Nov 2;12(21):2561. doi: 10.3390/cells12212561 (PMC10648219; doi:10.3390/cells12212561)
Supplement: Supplementary file 1 [file cells-12-02561-s001.zip › cells-2648750-supplementary.pdf]

Supplementary

Supplementary Table S1: Specific primer sequences.

mRNA	Forward Sequence	Reverse Sequence	Gene bank ID
Anp	TCCCGTATACAGTGCGGTGTC	GGAGGCATGACCTCATCTTC	NM_012612
Bnp	ACAATCCACGATGCAGAAGC	GAAGGCGCTGTCTTGAGACC	NM_031545
Nox2	TGTGGCTGTGATAAGCAGGAG	AATCCCAGCTCCCACTAACATC	NM_023965
Col1a1	CTGCACGAGTCACACCGGAA	CCAATGTCCAAGGGAGCCAC	NM_053304
Col3a1	TGGCTGCACTAAACACACTG	CCAATGTCATAGGGTGCGAT	NM_032085
Lox	CGGTTACTTCCAGTACGGTCTC	CCGCCCTATATGCTGAACTG	NM_017061
TGF-β1	CAGTGGCTGAACCAAGGAGAC	CTCGACGTTTGGGACTGATCC	NM_021578
Polr2a	GGTATTGAGCAGATCAGCAAGG	CAATGCCCAGTACCGTGAAG	XM_343922
Rpl-32	GGTGAAGCCCAAGATCGTCAA	TCTGGGTTTCCGCCAGTTTC	NM_013226.2
